# Bayesian Spatio-Temporal Modeling of *Schistosoma japonicum* Prevalence Data in the Absence of a Diagnostic ‘Gold’ Standard

**DOI:** 10.1371/journal.pntd.0000250

**Published:** 2008-06-11

**Authors:** Xian-Hong Wang, Xiao-Nong Zhou, Penelope Vounatsou, Zhao Chen, Jürg Utzinger, Kun Yang, Peter Steinmann, Xiao-Hua Wu

**Affiliations:** 1 National Institute of Parasitic Diseases, Chinese Center for Disease Control and Prevention, Shanghai, People's Republic of China; 2 Department of Public Health and Epidemiology, Swiss Tropical Institute, Basel, Switzerland; 3 Department of Disease Control, Ministry of Health, Beijing, People's Republic of China; 4 Jiangsu Institute of Parasitic Diseases, Wuxi, People's Republic of China; London School of Hygiene & Tropical Medicine, United Kingdom

## Abstract

**Background:**

Spatial modeling is increasingly utilized to elucidate relationships between demographic, environmental, and socioeconomic factors, and infectious disease prevalence data. However, there is a paucity of studies focusing on spatio-temporal modeling that take into account the uncertainty of diagnostic techniques.

**Methodology/Principal Findings:**

We obtained *Schistosoma japonicum* prevalence data, based on a standardized indirect hemagglutination assay (IHA), from annual reports from 114 schistosome-endemic villages in Dangtu County, southeastern part of the People's Republic of China, for the period 1995 to 2004. Environmental data were extracted from satellite images. Socioeconomic data were available from village registries. We used Bayesian spatio-temporal models, accounting for the sensitivity and specificity of the IHA test via an equation derived from the law of total probability, to relate the observed with the ‘true’ prevalence. The risk of *S. japonicum* was positively associated with the mean land surface temperature, and negatively correlated with the mean normalized difference vegetation index and distance to the nearest water body. There was no significant association between *S. japonicum* and socioeconomic status of the villages surveyed. The spatial correlation structures of the observed *S. japonicum* seroprevalence and the estimated infection prevalence differed from one year to another. Variance estimates based on a model adjusted for the diagnostic error were larger than unadjusted models. The generated prediction map for 2005 showed that most of the former and current infections occur in close proximity to the Yangtze River.

**Conclusion/Significance:**

Bayesian spatial-temporal modeling incorporating diagnostic uncertainty is a suitable approach for risk mapping *S. japonicum* prevalence data. The Yangtze River and its tributaries govern schistosomiasis transmission in Dangtu County, but spatial correlation needs to be taken into consideration when making risk prediction at small scales.

## Author Summary

Schistosomiasis is a serious public health problem in the People's Republic of China and elsewhere, and mapping of risk areas is important for guiding control interventions. Here, a 10-year surveillance database from Dangtu County in the southeastern part of the People's Republic of China was utilized for modeling the spatial and temporal distribution of infections in relation to environmental features and socioeconomic factors. Disease surveillance was done on the basis of a serological test, and we explicitly considered the imperfect sensitivity and specificity of the test when modeling the ‘true’ infection prevalence of *Schistosoma japonicum*. We then produced a risk map for *S. japonicum* transmission, which can assist decision making for local control interventions. Our work emphasizes the importance of accounting for the uncertainty in the diagnosis of schistosomiasis, and the potential of predicting the spatial and temporal distribution of the disease when using a Bayesian modeling framework. Our study can therefore serve as a template for future risk profiling of neglected tropical diseases studies, particularly when exploring spatial and temporal disease patterns in relation to environmental and socioeconomic factors, and how to account for the influence of diagnostic uncertainty.

## Introduction

Schistosomiasis japonica is a zoonotic disease caused by the digenetic trematode *Schistosoma japonicum*. Historically, the disease was endemic in 12 provinces of the People's Republic of China, with more than 10 million individuals infected [1]–[3]. Sustained control efforts implemented over the past 50 years have confined *S. japonicum* to seven provinces and brought down the number of infected people to less than 1 million [1]–[3]. The mean infection intensity has also decreased significantly [2]. However, surveillance and interventions are still warranted in 435 counties according to the 2005 annual report on the epidemiologic status of schistosomiasis in the People's Republic of China [4].

Geographic information system (GIS) and remote sensing technologies are increasingly utilized for risk mapping and prediction of schistosomiasis [5],[6]. Over the past decade, several studies have explored the relationship between the occurrence of schistosomiasis, its intermediate host snails and environmental factors, particularly land surface temperature (LST) and normalized difference vegetation index (NDVI) [7]–[14]. Socioeconomic factors and water contact patterns were also studied [11], [15]–[18]. The flexibility in modeling and parameter estimation renders Bayesian spatial modeling particularly attractive for risk factor analysis and mapping [19]–[21]. Early statistical methods employed for data analysis followed independent rather than spatially-correlated approaches. More recently, spatial modeling using Bayesian Markov chain Monte Carlo (MCMC) simulation-based inference has been applied to estimate the relation between environmental predictors, socioeconomic factors, and schistosomiasis. This approach allows the prediction of the prevalence and intensity of infection at non-sampled locations, taking into account the spatial correlation present in the data [11], [12], [21]–[25].

However, none of the above-mentioned studies pertaining to the spatial or spatio-temporal distribution of disease risk has taken into account the uncertainty of the diagnostic technique. In the case of schistosomiasis japonica, both serological (e.g., enzyme-linked immunosorbent assay (ELISA), indirect hemagglutination assay (IHA) [26]) and parasitological methods (e.g., Kato-Katz technique [27], miracidium hatching test [28]) are used in epidemiologic surveys. None of these diagnostic approaches has 100% sensitivity, however [28]–[31]. Although an enhanced sampling effort (e.g., multiple stool examinations) and simultaneous use of different diagnostics improve the sensitivity [32],[33] this strategy is not feasible in routine surveys due to logistic and financial constraints. In the early 1990s, the Chinese schistosomiasis control programme embarked on a two-pronged diagnostic approach. Local residents in *S. japonicum*-endemic areas are first screened with a serological test, followed by stool examination of seropositive individuals [29]. According to expert opinion, the sensitivity of ELISA ranges from 90% to 95%, and the specificity from 85% to 90%. In the case of the Kato-Katz technique, the estimated sensitivity and specificity are 20–70%, and 95–100%, respectively [32].

In this study, we employed a Bayesian approach to investigate the spatio-temporal patterns of *S. japonicum* infection, and to identify environmental and socioeconomic risk factors. In our models, we explicitly took into account the diagnostic uncertainty.

## Materials and Methods

### Study area

The study was carried out in Dangtu, one of 14 *S. japonicum*-endemic counties in Anhui province, southeastern part of the People's Republic of China. The first local case of schistosomiasis japonica was confirmed in 1953. Dangtu is situated on the lower reaches of the Yangtze River and stretches from 118°22′ to 118°53′E longitude and from 31°17′ to 31°42′N latitude. All three commonly recognized *S. japonicum* ecotypes are found in Dangtu, i.e., (i) plains with waterway networks, (ii) marshlands and lakes, and (iii) hilly and mountainous regions.

### Data sources

*S. japonicum* prevalence data were obtained from the annual county reports, covering the period from 1995 to 2004. Each year in September, field teams of the schistosomiasis control station in Dangtu sampled and surveyed the 114 schistosome-endemic villages as part of the national control program of schistosomiasis, which was approved by the institutional review board of the National Institute of Parasitic Diseases, Chinese Center for Disease Control and Prevention in Shanghai. The sampling frequency was in accordance with the prior classification of the respective village. Hence, villages with ongoing transmission were surveyed annually, villages where transmission was under control (prevalence <1%) were sampled every 2–3 years, and villages which had reached the criteria for transmission interruption (no human or animal cases within the past 5 years, no intermediate host snails observed in the previous year) were only surveyed if new snail habitats had been identified. During the 10-year surveillance period covered here, between 43 (in 1999 and 2002) and 68 (in 1998) villages were surveyed annually (median: 49). In sampled villages, all residents aged 5 to 65 years were invited to participate. One of the study requirements was that at least 80% of the eligible individuals should be tested. A two-pronged diagnostic approach was adopted; individuals were first screened by the IHA, followed by stool examination of seropositives. Parasitological diagnosis usually relied on the Kato-Katz technique [27]. Those found with *S. japonicum* eggs in their stool were treated with praziquantel. The median number of IHA tests performed per village was 778 (lower and upper quintiles: 302 and 1250). In this study, data from the Kato-Katz thick smear examinations were not used for further analysis, since some of the seropositives were not followed-up by the Kato-Katz technique due to recent treatments with praziquantel, and lack of compliance.

**Figure 1:**
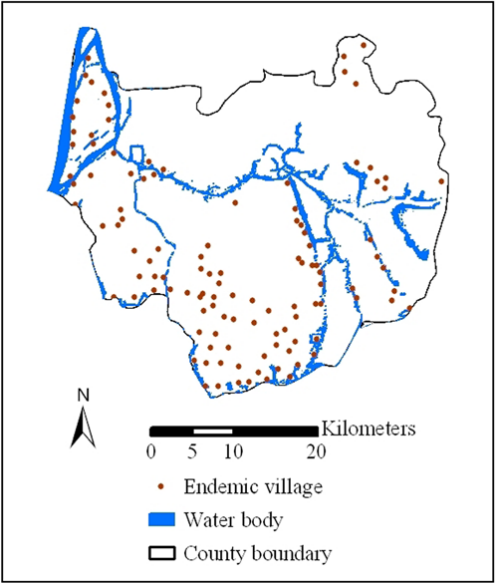
Location of the114 *S. japonicum*-endemic villages and the identified water bodies in Dangtu county, Anhui province, southeastern part of the People's Republic of China in 2004.

The geographic coordinates of the village committee houses in the *S. japonicum*-endemic villages were collected using hand-held global positioning system (GPS) receivers (Garmin Corp.; Olathe, KS, USA) and used as a proxi for the location of the village. Figure 1 shows the 114 *S. japonicum*-endemic villages in Dangtu county in relation to identified water bodies. Most endemic villages are located in the vicinity of water bodies or in the marshlands. Only four villages are situated in the northeastern hilly and mountainous region.

A SPOT5 image with a spatial resolution of 2.5 m and covering the whole study area, taken on February 9, 2004, was purchased from China Remote Sensing Satellite Ground Station (Beijing, People's Republic of China). This image was chosen because of its high quality (e.g., cloud cover <10%). With regard to water bodies, no major changes occurred from 1995 to 2004. Water bodies were identified using an unsupervised classification function of ERDAS IMAGINE version 8.6 (ERDAS LLC.; Atlanta, GA, USA). The shortest straight-line distance between each village and the closest water body was calculated in ArcGIS version 8.3 (ESRI; Redlands, CA, USA). For each year, one cloud-free Landsat-5 TM image with a spatial resolution of 30 m was purchased from China Remote Sensing Satellite Ground Station, covering the period from 1995 to 2004 (4 scenes were acquired in April, 3 in March, 2 in June, and 1 in August). LST and NDVI were extracted using the tools offered by ERDAS (http://gi.leica-geosystems.com). For each scene, the mean LST and NDVI within a 2-km buffer zone around the centroids of the study villages were calculated in ArcGIS.

Village-specific socioeconomic data were obtained from the annually-updated village registries. The available indices included annual average per-capita income and the proportion of households with tap water and improved sanitation.

Dangtu county was partitioned into 0.25×0.25 km grid cells for the generation of a smooth prediction map for 2005. The minimum distance from each grid cell centroid to the nearest water body was calculated in ArcGIS. For each cell, the mean LST and the mean NDVI were extracted from the 2005 Landsat scene.

### Statistical analysis

LST and NDVI data were standardized by subtracting the arithmetic mean calculated from data within a 2-km buffer zone around the centroids of the study villages for each scene and then dividing the standard deviation using SAS version 8.0 (SAS Institute Inc.; Cary, NC). Villages were stratified into five wealth quintiles, based on the annual average per-capita income. The relationship between *S. japonicum* seroprevalence and village-specific environmental and socioeconomic surrogate measures was examined using scatter plots.

### Bayesian spatio-temporal modeling

A Bayesian approach was utilized to explore the spatio-temporal patterns of the *S. japonicum* seroprevalence data. The relationship between seroprevalence and environmental and socioeconomic covariates was also examined. We applied two different model specifications. The first set of models assumed no diagnostic error of the IHA. The second set of models explicitly took into account the diagnostic error, thus correcting for the estimated ‘true’ sensitivity and specificity of the IHA. For 2005, we created a smoothed predictive map of the *S. japonicum* prevalence.

### Seroprevalence of *S. japonicum* in the absence of a diagnostic ‘gold’ standard

Let *n_it_* and *z_it_* be the number of examined and positive subjects by IHA, respectively, of village *i* (*i* = 1,…,*N*) in year *t* (*t* = 1,…,*T*). We assumed that *z_it_* follows a binomial distribution, that is *z_it_* ∼ Binomial(*p_it_*,*n_it_*), where *p_it_* is the seroprevalence following the standard formulation of the logistic regression model. We introduced covariate effects on the logit transformation of *p_it_*, that is 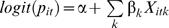, where α is the intercept, β*_k_* denotes a regression coefficient, and *X_itk_* is the environmental or socioeconomic covariate.

The standard assumption of this formulation is that the observations are independent. However, our data are spatially correlated because common environmental factors concurrently influence the infection risk in neighboring villages. Similarly, the data are temporally correlated because they have been obtained through repeated cross-sectional surveys. Ignoring these correlations, we would overestimate the significance of the covariates. To account for the spatio-temporal correlation, we introduced village-specific and year-specific random effects, *u_i_* and *v_t_*, respectively, as follows: 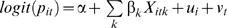. We defined a latent stationary and isotropic spatial process [34] on *u_i_*, by assuming that *u* = (*u*_1_,*u*_2_,…,*u_N_*)*^T^* has a multivariate normal distribution with variance-covariance matrix Σ, that is, *u*∼*MVN*(0,Σ). We defined Σ by an exponential correlation function, i.e., Σ*_lm_* = σ^2^exp(−ϕ*d_lm_*), where *d_lm_* is the shortest straight-line distance between villages *l* and *m*, σ^2^ models the geographic variability, and ϕ is a smoothing parameter controlling the rate of decline of the spatial correlation with distance throughout the study period. For the exponential correlation function we have adopted the minimum distance at which correlation becomes less than 5%, which is defined by 3/ϕ and expressed in meters. Similar to previous spatio-temporal modeling of schistosomiasis [12], we defined a first-order autoregressive process (AR(1)) on *v_t_*, assuming that temporal correlation ρ exists only with the preceding year [35].

An alternative spatio-temporal structure was modeled by assuming that spatial correlations evolve over time (space-time interaction) that is 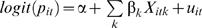, where *u_t_* = (*u*_1*t*_,*u*_2*t*_,…,*u_Nt_*)*^T^* is the spatio-temporal random effect such that 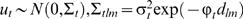 with the parameter ϕ*_t_* controlling the rate of decline of spatial correlation with distance in year *t*. We assessed the significance of covariates by including only environmental, or only socioeconomic, or both types of covariates.

### Seroprevalence of *S. japonicum* taking into account the diagnostic error

The model detailed before was based on the assumption that the IHA reliably diagnoses a *S. japonicum* infection, i.e., its sensitivity and specificity are 100%. However, since IHA and other diagnostic tests have shortcomings [29], we made an attempt to incorporate the diagnostic error of IHA into our modeling framework.

Expert opinions on the diagnostic performance of IHA were gathered by means of a questionnaire survey, as described elsewhere [32]. The experts' consensus was that the sensitivity and specificity of IHA is 80–95% and 70–80%, respectively. These values were fed into the model as prior information.

Let π*_it_* be the underlying true prevalence of *S. japonicum* infection for village *i* in year *t*, and *p_it_* the observed prevalence of infection. Following the model specifications of Booth and colleagues [33] and Wang et al. [32], we assumed that the number of seropositives *z_it_* has a binomial distribution that is *z_it_* ∼ Binomial(*p_it_*,*n_it_*), and related the observed and true prevalence via the equation *p_it_* = π*_it_s_jt_* + (1−π*_it_*)(1−*c_jt_*). This equation is derived from the law of total probability, where *s_jt_* and *c_jt_* are the sensitivity and specificity of IHA for village *j* (*j* = 1,…,*J*) in year *t*, respectively, where *j* is a group of adjacent villages. The models described previously were fitted, but with underlying prevalence π*_it_* instead of the seroprevalence *p_it_*.

### Model validation and comparison

The same database was used throughout the study. We randomly selected 93 out of the 114 *S. japonicum*-endemic villages (82%), and used the surveys conducted between 1995 and 2004 for fitting the models, employing 408 out of the available 508 surveys. The remaining 100 surveys carried out in the other 21 villages over the same period served for model validation. In a first step, we compared the goodness-of-fit of the models by using the deviance information criterion (DIC) [36]. The model with the smallest DIC value was considered the best-fitting one. Next, we evaluated the predictive abilities of different models by calculating five different Bayesian credible intervals (BCIs) with probability coverage equal to 5%, 25%, 50%, 75%, and 95% of the posterior predictive distribution at the test locations, as suggested elsewhere [19]. Models with a high percentage of records falling into the narrowest BCIs were considered to have good predictive abilities.

Following a Bayesian model formulation, we adopted vague normal prior distributions for each regression coefficient β*_k_* and intercept α, vague inverse gamma priors for variances, and a uniform prior ranging from −1 to 1 for temporal correlation ρ. Informative beta prior distributions derived from expert opinion that is, beta (67.18, 9.60) and beta (224.25, 74.75), were used for sensitivity *s_jt_* and specificity *c_jt_*, respectively. We assumed that the prior for the spatial correlation ranged from 0.01 to 0.99 at the minimal distance between villages (0.6 km) and from 0 to 0.2 at maximal distance (49 km), thus uniform priors ranging from 0.017 to 7.675 were used for the spatial decay parameters ϕ and ϕ*_t_*. Two-chain MCMC was used for parameter estimation. Model convergence was assessed by visually inspecting the time series plot for each parameter, and Gelman-Rubin statistics [37]. The inference of the parameters was based on 15,000 iterations of both chains after the burn-in phase. Model fit was carried out in WinBUGS 1.4.1 (Imperial College and MRC, London, UK).

## Results

### *S. japonicum*-endemic villages

**Figure 2:**
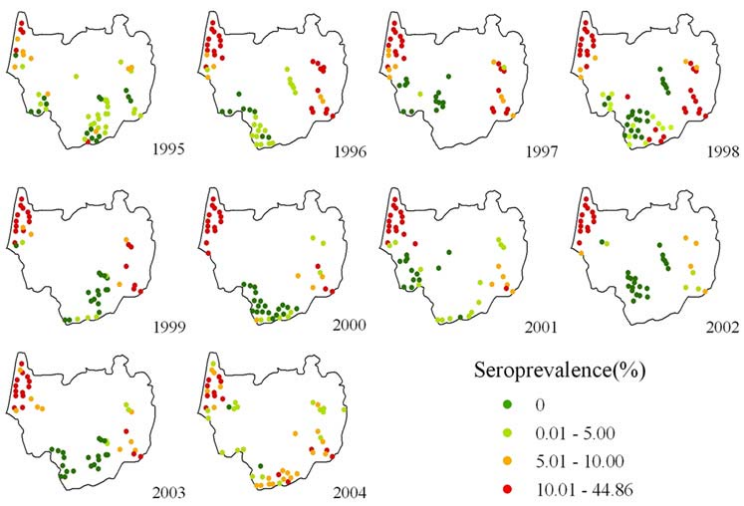
*S. japonicum* seroprevalences in the 114 surveyed villages in Dangtu county, Anhui province, southeastern part of the People's Republic of China, from 1995 to 2004.

Figure 2 shows the observed seroprevalence in the study villages, according to survey year. Commonly, high seroprevalences were observed in villages located in close proximity to large rivers. In 27% of the village surveys the seroprevalence was zero, whereas a mean seroprevalence ≥10% was found in 41% of the surveys.

### Model outcomes in the absence of a diagnostic ‘gold’ standard

Table 1 summarizes the goodness-of-fit and the predictive ability of the models which did not take into account the diagnostic error of IHA. The smaller DIC values of the spatio-temporal models indicate that they fitted the data better than the non-spatial ones. The predictive ability of the models could be improved significantly by considering spatio-temporal random effects. Moreover, the percentage of testing records falling into smaller BCIs of the posterior predictive distribution was considerably higher in the spatio-temporal models than in the non-spatial ones. Models considering the temporal evolution of spatial correlation also appeared to better fit the data than those assuming independent spatial and temporal processes. Considering also socioeconomic information did not further improve the model. Hence, the model with environmental covariates and variable spatial correlation was considered the best-fitting one.

**Table 1:** The number of testing records falling in the 5%, 25%, 50%, 75%, and 95% BCIs of the posterior predictive distribution and the corresponding DIC value when modeling *S. japonicum* seroprevalence without taking into account the diagnostic error.

Model specification	Percentage falling in					DIC
	5% BCI	25% BCI	50% BCI	75% BCI	95% BCI	
All covariates, non-spatial	1	3	3	7	16	24,316
All covariates, spatio-temporal 1	10	30	50	74	87	9,823
Socioeconomic, spatio-temporal 1	9	25	47	72	87	9,911
Environmental, spatio-temporal 1	12	28	42	68	92	10,624
All covariates, spatio-temporal 2	27	53	73	89	97	2,431
Socioeconomic, spatio-temporal 2	28	44	68	86	96	2,434
Environmental, spatio-temporal 2	31	56	76	89	97	2,428

Socioeconomic: only socioeconomic covariates included.

Environmental: only environmental covariates included.

Spatio-temporal 1: independent spatial and temporal random effects assumed.

Spatio-temporal 2: spatial correlations evolving over time assumed.

### Model outcomes when accounting for the diagnostic error

As shown in Table 2, incorporating the sensitivity and specificity of IHA as model parameters, resulted in smaller DIC values in the annual differences in the spatial correlation. When models also considered socioeconomic information there was no further improvement. Actually, the percentages of testing records falling into smaller BCIs were larger in a similar model that only considered environmental covariates. Thus, the model without explicit consideration of socioeconomic data was considered the best-fitting one. However, its predictive ability was inferior to that of the best-performing model which did not take into account the diagnostic error of IHA (4% *versus* 31% of the test records falling into the 5% BCI).

**Table 2:** The number of testing records falling in the 5%, 25%, 50%, 75%, and 95% BCIs of the posterior predictive distribution and the corresponding DIC value when modeling the underlying ‘true’ prevalence of *S. japonicum* infection.

Model specification	Percentage (%) falling in					DIC
	5% BCI	25% BCI	50% BCI	75% BCI	95% BCI	
All covariates, non-spatial	1	5	12	15	25	9,891
All covariates, spatio-temporal 1	2	6	17	26	42	8,463
Socioeconomic, spatio-temporal 1	3	9	17	23	36	8,883
Environmental, spatio-temporal 1	1	7	16	21	35	9,002
All covariates, spatio-temporal 2	1	10	17	32	49	7,180
Socioeconomic, spatio-temporal 2	0	11	20	29	45	7,191
Environmental, spatio-temporal 2	4	10	18	37	51	7,184

Socioeconomic: only socioeconomic covariates included.

Environmental: only environmental covariates included.

Spatio-temporal 1: independent spatial and temporal random effects assumed.

Spatio-temporal 2: spatial correlations evolving over time assumed.

### Relationship between *S. japonicum* infection and covariates

Table 3 summarizes the results of the best-fitting spatio-temporal models regarding the observed *S. japonicum* seroprevalence and the ‘true’ infection prevalence. The prevalence increased with the mean LST (regression coefficients: 0.201 and 0.669 for seroprevalence and ‘true’ infection prevalence, respectively), and was negatively correlated with the mean NDVI (regression coefficient: −0.327 and −1.044, respectively). The seroprevalence was also inversely related to the distance to the closest water body (regression coefficient: −0.277 and −1.069, respectively). The estimated variances using the model with adjusting for the diagnostic error were larger, as suggested by larger 95% BCIs. The relationship between the serostatus and socioeconomic covariates was not further explored since the selected variables neither improved the goodness-of-fit nor the prediction ability of the models.

**Table 3:** Bayesian hierarchical logistic model regression coefficients (posterior median with 95% BCI in brackets) in the best-fitting models[Table-fn nt109] when modeling seroprevalence and underlying ‘true’ prevalence of *S. japonicum* infection, respectively.

Parameter (variable)	Modeling seroprevalence	Modeling underlying prevalence^a^
α (intercept)	−3.180 (−3.567, −2.736)	−8.053 (−8.836, −6.876)
β1 (LST mean)	0.201 (0.086, 0.337)	0.669 (0.270, 1.116)
β2 (NDVI mean)	−0.327 (−0.479, −0.176)	−1.044 (−1.549, −0.651)
β3 (distance to water body)	−0.277 (−0.435, −0.112)	−1.069 (−1.770, −0.353)
ϕ1 (spatial decay 1995)	0.505 (0.169, 6.549)	3.904 (0.701, 7.493)
ϕ2 (spatial decay 1996)	0.144 (0.050, 0.439)	3.554 (0.522, 7.481)
ϕ3 (spatial decay 1997)	0.091 (0.031, 0.251)	3.721 (0.555, 7.474)
ϕ4 (spatial decay 1998)	0.265 (0.119, 0.632)	3.893 (0.556, 7.486)
ϕ5 (spatial decay 1999)	0.149 (0.052, 1.537)	0.801 (0.145, 7.170)
ϕ6 (spatial decay 2000)	0.089 (0.033, 0.252)	1.317 (0.121, 7.342)
ϕ7 (spatial decay 2001)	0.221 (0.072, 5.326)	4.544 (1.005, 7.513)
ϕ8 (spatial decay 2002)	0.057 (0.021, 0.181)	4.152 (0.664, 7.507)
ϕ9 (spatial decay 2003)	0.054 (0.021, 0.143)	4.047 (0.672, 7.488)
ϕ10 (spatial decay 2004)	0.363 (0.138, 4.599)	4.487 (1.016, 7.515)

Best-fitting models: spatial correlations evolving over time assumed and only environmental covariates included.

a Adjusted for diagnostic error of IHA.

### Spatio-temporal pattern of *S. japonicum* infection

**Figure 3:**
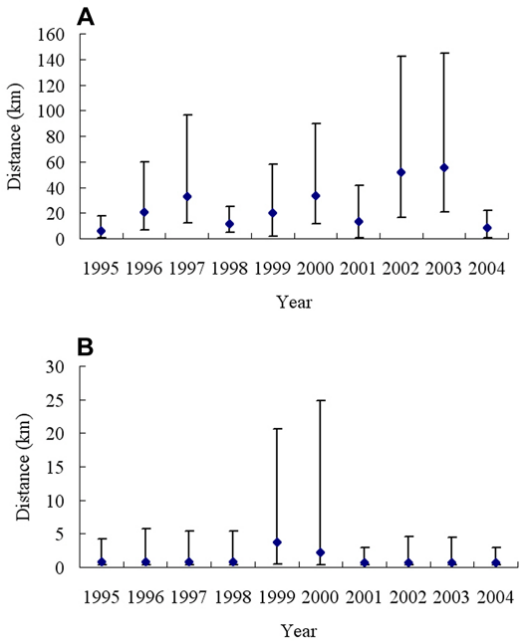
The minimum distance (posterior median and 95% BCI) at which spatial correlation was less than 5% in Dangtu county, Anhui province, southeastern part of the People's Republic of China from 1995 to 2004. (A) For seroprevalence (diagnostic error ignored); (B) for underlying prevalence (diagnostic error taken into account).

The best-fitting spatio-temporal models indicated that the spatial correlation structures of the observed seroprevalence and the ‘true’ prevalence differed from one year to another, albeit not significantly (Table 3). Generally, the spatial correlation of the seroprevalence declined at a slower pace than that of the ‘true’ prevalence (smaller values of the parameter ϕ indicate a slower decay of the correlation with distance). For the measured seroprevalence, the shortest distance at which the spatial correlation was below 5% was determined in 1995 (5.9 km; 95% confidence interval (CI): 0.5–17.8 km). The maximum value of 55.6 km (95% CI: 21.0–144.4 km) was modeled for 2003. For the underlying ‘true’ prevalence, the respective distances were 0.7 km (95% CI: 0.4–3.0 km in 2001) and 3.7 km (95% CI: 0.4–20.7 km in 1999; Figure 3). The model for the measured seroprevalence further indicated a fast decline of the spatial correlation with distance in 1995, 1998, and 2001, and a slower decay over the respective ensuing two years.

### Prediction of the ‘true’ *S. japonicum* prevalence in 2005

**Figure 4:**
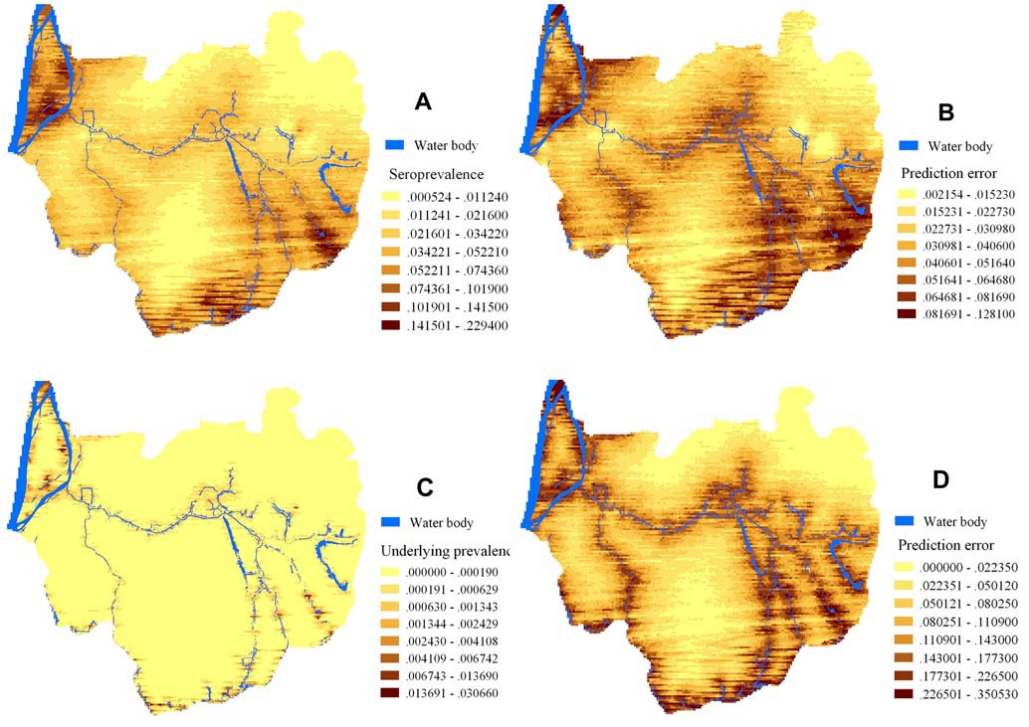
Prevalence maps of *S. japonicum* infection in Dangtu county, Anhui province, southeast China in 2005. (A) Map of predicted prevalence, and (B) map of prediction error when diagnostic error is ignored; (C) Map of predicted prevalence, and (D) map of prediction error when diagnostic error is considered.

The *S. japonicum* prevalence in Dangtu county was predicted for 2005, based on the spatial correlation structures observed in the preceding year. The predicted seroprevalence in the county ranged from 0.05% to 22.9% (posterior median). Most of the predicted high-seroprevalence areas are located in close proximity to water bodies, especially the Yangtze River, and in the southeast of the county (data not shown). The predicted ‘true’ *S. japonicum* prevalence ranged from nil to 3.7% (posterior median). The locations for which a relatively high ‘true’ prevalence was predicted are again located in the vicinity of water bodies (Figures 4a and 4c). The distribution of the prediction error is depicted in Figures 4b and 4d.

## Discussion

In this study, we estimated the ‘true’ *S. japonicum* prevalence in a schistosome-endemic county of the People's Republic of China by explicitly taking into consideration the diagnostic error of a widely used serological test, i.e. IHA. Additionally, we explored the spatial distribution over time, and produced a predictive risk map for the year 2005. Since antibody-based immunological tests, such as IHA and ELISA, cannot distinguish between an active and a recently cleared infection, these techniques result in low specificity in areas where chemotherapy is provided on a regular basis [31]. Thus, the analysis of uncorrected seroprevalence data would only be suggestive of the overall infection pressure [38]. In order to better understand the epidemiologic characteristics of schistosomiasis japonica, we accounted for the lack of sensitivity and specificity of the standard serological test employed in our study setting by using a Bayesian approach, and compared the outcome with that of the uncorrected model that assumed 100% sensitivity and specificity.

In recent years, significant progress has been made with Bayesian spatio-temporal models. Thus our understanding of the epidemiology of infectious diseases in general [39],[40], and schistosomiasis in particular [22], has been improved. We used two types of spatio-temporal models; one assumed independent spatial and temporal random effects, and the second assumed that spatial correlations evolved over time (space-time interaction). Similar approaches have been successfully employed before [12],[41],[42]. We considered a stationary spatial process, although recent investigations suggest that non-stationarity is a more reasonable approach [19],[21]. The reasons were as follows. First, Dangtu county is small, spanning 50 km at most. Second, the local environment in this setting is rather uniform, and the study area mainly consists of plain regions with waterways, marshlands and lakes. In future analyses, it would be interesting to investigate anisotropic processes.

Remotely-sensed environmental data are increasingly utilized in schistosomiasis research [5],[11],[43],[44]. Temperature and vegetation coverage are among the most frequently investigated environmental features, as they can be readily derived from satellite images. Their utility for an enhanced understanding of the local epidemiology of schistosomiasis has been demonstrated extensively [8],[11],[44]. In this study, LST and NDVI were extracted from Landsat-5 TM images, and averaged values for each village for individual survey years were calculated for 2-km buffer zones around the centroid of each village. The 2-km buffer zone approximately corresponds to an average village in Dangtu, and most daily activities take place within such a range. Prevailing weather conditions did not allow us to obtain all remotely-sensed data in the same month, i.e., April, the first month of the local transmission season [12]. To remedy this issue, we standardized the indices.

Three important findings emerged from our study. First, LST was positively associated with *S. japonicum* prevalence, whereas the NDVI and distance to water bodies were negatively associated. These observations are consistent with previous findings [12],[23]. However, the non-spatial models revealed that the prediction ability of these covariates was poor whether or not the diagnostic error of IHA was taken into account. It is thus conceivable that the environmental factors explained the local *S. japonicum* prevalence to a small degree only. The effects of socioeconomic factors such as the annual average per-capita income, the proportion of households with piped water supply, and the proportion of households with access to improved sanitation were even smaller, contrasting results for *S. mansoni* in Côte d'Ivoire [11],[45]. Possible explanations for this finding are that socioeconomic factors could be disconnected from the epidemiology of schistosomiasis at small spatial scales, and improved water supply and sanitation do not necessarily change the water contact pattern of villagers [15]. A model incorporating socioeconomic variables measured at the individual level rather than at the village level as done here, might result in a better fit.

Second, the spatial correlation of the seroprevalence and the estimated ‘true’ prevalence of *S. japonicum* occurred over greater distances for the former than the later. Our study is the first to compare the range of spatial correlation of the seroprevalence with that of the underlying prevalence. Additional investigations in different settings are warranted to verify this finding and explore possible reasons. Spatial correlation has also been documented for *S. haematobium* and *S. mansoni* in different African settings [11],[23]. The importance of the spatial correlation was underscored by the finding that the predictive ability of the model was greatly improved when spatio-temporal random effects were incorporated. The inclusion of the uncertainty about IHA sensitivity and specificity lowered the predictive ability, and increased the prediction errors since additional sources of errors were considered and the spatial correlation occurred over shorter distances. Whilst the spatial correlation varied from one year to another, no strong temporal trend was observed in our study. One possible reason is that the duration of our inquiry (i.e., 10 years) is not long enough for capturing prevailing temporal patterns.

Third, smoothed risk maps for 2005 were created based on the spatial correlation found in the preceding year. Since no significant temporal trend was detected from 1995 to 2004, it was decided to use the most recent data only. It is evident that most human infections were predicted to occur in close proximity to the Yangtze River and its tributaries. It has already been noted before that *Oncomelania hupensis* in the waterways connected to the Yangtze River are difficult to eliminate, and that snails can readily re-colonize cleared areas [2]. The prediction maps highlighted the areas (villages) at high risk of *S. japonicum* infection, and emphasized the important role of the Yangtze River in the transmission of schistosomiasis in Dangtu county. Implications for the local schistosomiasis control program are that control measures should be targeted to those villages at highest risk.

One limitation of our study is that about 20% of the eligible population (aged 5–65 years) in the sampled villages was not surveyed. It is hard to predict whether non-compliance biased our risk profiles. Another limitation is that non-surveyed villages were excluded from the analysis in the corresponding year(s) and their effects on the estimates were not taken into consideration in the models, since there might be too many parameters to be estimated.

In conclusion, we have presented an in-depth study on the spatio-temporal pattern of *S. japonicum* within a single county. Importantly, we explicitly took into account the diagnostic error of the serological screening test, and employed a Bayesian modeling approach, through which the underlying ‘true’ prevalence of *S. japonicum* infection could be estimated and predicted. There is considerable spatial correlation and annual variability of *S. japonicum* infection. Hence, for small-scale prediction, accounting for the spatial correlation seems more important than considering the risk factors included in our study. Finally, the Yangtze River and its tributaries play an essential role in the local epidemiology of schistosomiasis japonica.

## Supporting Information

Alternative Language Abstract S1 Translation of the abstract into Chinese by Xiao-Nong Zhou.
(0.05 MB PDF)

Alternative Language Abstract S2 Translation of the abstract into German by Peter Steinmann.
(0.03 MB DOC)
