# Supplementary material for: Bayesian Spatio-Temporal Modeling of Schistosoma japonicum Prevalence Data in the Absence of a Diagnostic ‘Gold’ Standard
Source: PLoS Negl Trop Dis. 2008 Jun 11;2(6):e250. doi: 10.1371/journal.pntd.0000250 (PMC2405951; doi:10.1371/journal.pntd.0000250)
Supplement: Alternative Language Abstract S1 — Translation of the abstract into Chinese by Xiao-Nong Zhou. (0.05 MB PDF) [file pntd.0000250.s001.pdf]

缺乏“金标准”时日本血吸虫流行率的贝叶斯时空模型的构建

摘要

背景: 空间模型已越来越多地应用于揭示人口、环境、社会经济因素与传染病流行率间的关系。然而仍缺少对于诊断不确定性分析的时空模型。

方法/主要结果: 我们收集了1995年至2004年中国当涂县114个血吸虫病流行村的日本血吸虫间接血凝试验人群阳性率。而环境数据来自卫星遥感图像，社会经济数据来自村级登记资料。基于总概率原则获得了间接血凝试验的敏感性与特异性，并应用贝叶斯时空模型以血清阳性率观察值推算出“真”值。结果发现，日本血吸虫感染危险度与地表温度呈正相关，与归一化植被指数及居住地至水体距离间呈负相关，与调查村的社会经济状况无显著相关性。各年间的日本血吸虫血清阳性率与估算感染率间的空间相关结构不尽相同。以诊断误差调整的模型所产生的变异较未调整模型的变异更大。2005年的预测图显示以往和当时的血吸虫感染主要发生在与长江相连的河流。

结论/意义: 加入诊断不确定性的贝叶斯时空模型，是实现利用日本血吸虫流行资料对高危区域制图的一个好方法。长江及通江河道是当涂县血吸虫病传播的主要区域，因此，当在小尺度上进行高危区域预测时需要加入空间相关性因素。
