# Supplementary material for: Bayesian Spatio-Temporal Modeling of Schistosoma japonicum Prevalence Data in the Absence of a Diagnostic ‘Gold’ Standard
Source: PLoS Negl Trop Dis. 2008 Jun 11;2(6):e250. doi: 10.1371/journal.pntd.0000250 (PMC2405951; doi:10.1371/journal.pntd.0000250)
Supplement: Alternative Language Abstract S2 — Translation of the abstract into German by Peter Steinmann. (0.03 MB DOC) [file pntd.0000250.s002.doc]

**Räumlich-zeitliche Modellierung von *Schistosoma japonicum* Prävalenzdaten mittels Bayes’scher Statistik in Abwesenheit eines diagnostischen Goldstandards**

Zusammenfassung

***Hintergrund:*** Räumliche Modellierung wird zunehmend eingesetzt zur Untersuchung der Beziehungen zwischen demographischen, Umwelt- und sozio-ökonomischen Faktoren einerseits, und der Prävalenz von Infektionskrankheiten andererseits. Allerdings gibt es nur wenige Studien, welche die Messunsicherheit von diagnostischen Techniken bei der räumlich-zeitlichen Modellierung berücksichtigen.

***Methoden/Wichtigste Ergebnisse:*** Wir entnahmen den Jahresberichten aus 114 *S. japonicum*-endemischen Dörfern im Bezirk Dangtu im Südosten der Volksrepublik China die Angaben zur jährlichen Prävalenz der Asiatischen Bilharziose zwischen 1995 und 2004. Diese Daten waren mittels eines standardisierten indirekten Hämagglutinations-Tests (IHT) ermittelt worden. Satellitenbilder lieferten Umweltdaten. Sozio-ökonomische Angaben aus Gemeinderegistern standen ebenfalls zur Verfügung. Um die Beziehung zwischen der beobachteten und der „wahren“ Prävalenz von *S. japonicum* zu bestimmen, entwickelten wir Bayes’sche räumlich-zeitliche Modelle, welche die Sensitivität und Spezifität von IHT mittels einer aus dem Gesetz der Gesamtwahrscheinlichkeit abgeleiteten Gleichung berücksichtigen. Das Risiko für Asiatische Bilharziose korrelierte positiv mit der mittleren Oberflächentemperatur des Bodens, und negativ mit dem mittleren normalisierten differenzierten Vegetationsindex und der Entfernung zum nächsten Gewässer. Wir fanden keine signifikante Beziehung zwischen *S. japonicum* und den sozio-ökonomischen Bedingungen in den untersuchten Dörfern. Die Struktur der räumlichen Korrelation zwischen der beobachteten *S. japonicum* Seroprävalenz und der geschätzten Infektionsprävalenz schwankte von Jahr zu Jahr. Die mittels eines den diagnostischen Fehler berücksichtigenden Modells geschätzten Varianzen überstiegen die Werte unkorrigierter Modelle. Wir erstellten eine Risikokarte für Asiatische Bilharziose im Jahr 2005. Diese Karte zeigte auf, dass die Mehrzahl der früheren wie auch der gegenwärtigen Infektionen in der Nähe des Jangtse Flusses auftreten.

***Schlussfolgerung/Bedeutung:*** Räumlich-zeitliche Modellierung mittels Bayes’scher Statistik und unter Berücksichtigung der diagnostischen Unsicherheit ist ein vielversprechender Ansatz, um auf Prävalenzdaten basierende *S. japonicum*-Risikokarten zu erstellen. Der Jangtse und seine Zuflüsse bestimmen die Übertragung der Asiatischen Bilharziose im Bezirk Dangtu, doch muss die räumliche Korrelation bei der kleinräumlichen Risiko-Voraussage mitberücksichtigt werden.

Übersetzung: Peter Steinmann
